# 100% Orange juice consumption is associated with better diet quality, improved nutrient adequacy, decreased risk for obesity, and improved biomarkers of health in adults: National Health and Nutrition Examination Survey, 2003-2006

**DOI:** 10.1186/1475-2891-11-107

**Published:** 2012-12-12

**Authors:** Carol E O’Neil, Theresa A Nicklas, Gail C Rampersaud, Victor L Fulgoni III

**Affiliations:** 1 Louisiana State University Agricultural Center, 261 Knapp Hall, Baton Rouge, Louisiana, 70803, USA; 2 USDA/ARS Children’s Nutrition Research Center, Department of Pediatrics, Baylor College of Medicine, Houston, Texas, 77030, USA; 3 Food Science and Human Nutrition Department, University of Florida, Gainesville, Florida, 32611, USA; 4 Nutrition Impact, LLC, Battle Creek, Michigan, 49014, USA

**Keywords:** Orange juice consumption, 100% fruit juice consumption, Diet quality, Nutrients, Nutrient adequacy, Adults, Weight, Obesity, Metabolic syndrome, NHANES

## Abstract

**Background:**

Consumption of 100% orange juice (OJ) has been positively associated with nutrient adequacy and diet quality, with no increased risk of overweight/obesity in children; however, no one has examined these factors in adults. The purpose of this study was to examine the association of 100% OJ consumption with nutrient adequacy, diet quality, and risk factors for metabolic syndrome (MetS) in a nationally representative sample of adults.

**Methods:**

Data from adults 19+ years of age (n = 8,861) participating in the National Health and Nutrition Examination Survey 2003-2006 were used. The National Cancer Institute method was used to estimate the usual intake (UI) of 100% OJ consumption, selected nutrients, and food groups. Percentages of the population below the Estimated Average Requirement (EAR) or above the Adequate Intake (AI) were determined. Diet quality was measured by the Healthy Eating Index-2005 (HEI-2005). Covariate adjusted logistic regression was used to determine if consumers had a lower odds ratio of being overweight or obese or having risk factors of MetS or MetS.

**Results:**

Usual *per capita* intake of 100% OJ was 50.3 ml/d. Among consumers (n = 2,310; 23.8%), UI was 210.0 ml/d. Compared to non-consumers, consumers had a higher (p < 0.05) percentage (% ± SE) of the population meeting the EAR for vitamin A (39.7 ± 2.5 vs 54.0 ± 1.2), vitamin C (0.0 ± 0.0 vs 59.0 ± 1.4), folate (5.8 ± 0.7 vs 15.1 ± 0.9), and magnesium (51.6 ± 1.6 vs 63.7 ± 1.2). Consumers were also more likely to be above the AI for potassium (4.1 ± 0.8 vs 1.8 ± 0.2). HEI-2005 was significantly (p < 0.05) higher in consumers (55.0 ± 0.4 vs 49.7 ± 0.3). Consumers also had higher intakes of total fruit, fruit juice, whole fruit, and whole grain. Consumers had a lower (p < 0.05) mean body mass index (27.6 ± 0.2 vs 28.5 ± 0.1), total cholesterol levels (197.6 ± 1.2 vs 200.8 ± 0.75 mg/dL), and low density lipoprotein-cholesterol levels (112.5 ± 1.4 vs 116.7 ± 0.93 mg/dL). Finally, compared to non-consumers of 100% OJ, consumers were 21% less likely to be obese and male consumers were 36% less likely to have MetS.

**Conclusion:**

The results suggest that moderate consumption of 100% OJ should be encouraged to help individuals meet the USDA daily recommendation for fruit intake and as a component of a healthy diet.

## Background

Consumption of 100% fruit juice (FJ) has been associated with higher intakes of key nutrients, including vitamins C and B-6, folate, thiamin, magnesium, and potassium, as well as better diet quality, and increased intake of total and whole fruit consumption in children [1-4] and adults [3] as compared to those that do not consume 100% FJ. There have been concerns about overweight/obesity in consumers of 100% FJ, especially children [5-7]. Most studies have been conducted in children and have not shown a relationship between 100% FJ consumption and weight [8]. Cross-sectional studies that have been conducted in adults have shown that consumption of 100% FJ has been inversely associated with body mass index (BMI) [9,10] and obesity [10]; however, the longitudinal Nurses’ Health Study II showed that 100% FJ consumption was positively associated with weight gain [11].

The relationship between consumption of 100% FJ and other markers of disease among adults is inconsistent. Pereira and Fulgoni [10], using data from the National Health and Nutrition Examination Survey (NHANES) 1999-2004 and Yoo et al. [12] using data from the Bogalusa Heart Study, showed no association of risk of metabolic syndrome (MetS) among 100% FJ consumers; another study showed that, in middle aged and older adults, fasting glucose, but not fasting insulin [13] was lower in 100% FJ consumers. In different studies, diabetes risk was shown to be associated with 100% FJ consumption [14] or not [11]. Data from the CARDIA study have shown an association of a reduced risk of hypertension with 100% FJ consumption [15]. Since data on the effects of consumption of 100% OJ on adult health are conflicting, further studies are needed.

Few studies have examined the effect of specific fruit juices on diet and health. Orange juice (OJ) is the most popular 100% FJ consumed in the US. In 2009, *per capita* availability of OJ was 14.84 liters [16]. Orange juice is also one of the most nutrient dense 100% FJ, regardless of type of density measures used in the evaluation [17]. Two hundred and thirty seven ml of 100% OJ provides 469 kilojoules (kJ) (112 kcal), 21 g total sugars, 124 mg vitamin C, 27 mg magnesium, 0.10 mg vitamin B-6, 74 μg Dietary Folate Equivalents, 496 mg potassium, and only 0.06 g saturated fatty acids (SFA) and 2.48 mg sodium [18]. Some brands of commercially available 100% OJ are fortified with fiber, calcium, or vitamin D; these have been identified as nutrients of public health concern in the 2010 Dietary Guidelines for Americans [19].

In vitro [20] and animal studies [21,22] have suggested that citrus juices or components of these juices, including the flavanones hesperidin and naringin (or their aglycone forms hesperetin and naringenin), may have beneficial effects on blood lipids. Clinical studies conducted in adults have shown that consumption of 100% OJ has been associated with health benefits including positive effects on blood lipids [23-26]—especially in hypercholesterolemic individuals, lower levels of several oxidative or inflammatory stress biomarkers [27-29], and lower blood pressure [30]. Epidemiologic studies, using a nationally representative sample, looking at the association between consumption of 100% OJ and health markers are lacking. The objective of this study was to examine the association of 100% OJ usual intakes (UI) on select nutrients, food group equivalents, diet quality, weight parameters, and risk factors associated with cardiovascular disease and metabolic syndrome in adults.

## Methods

### Study population

Data from adults 19+ yrs (n = 8,861) participating in the NHANES 2003-2006 were combined for these analyses to increase the sample size. Females were excluded from the study if they were pregnant or lactating. Demographic information [31] and physical activity levels [32] were determined from the NHANES interview. NHANES has stringent consent protocols and procedures to ensure confidentiality and protection from identification [33]. Since this was a secondary data analysis with a lack of personal identifiers, this study was exempted by the Louisiana State University Agricultural Center Institutional Review Board.

### Determination of dietary intake data

Dietary data were collected using two 24-hour dietary recalls using an automated multiple-pass method [34,35]; the first recall was conducted in person by a trained interviewer and the second recall was conducted 3-10 days later via telephone. Only recall data judged to be complete and reliable by the National Center for Health Statistics staff were included in this study. Detailed descriptions of the dietary recalls and data collection are available in the NHANES Dietary Interviewer’s Training Manual [36].

In this study, 100% OJ was defined using the United States Food and Drug Administration definition [37] for 100% FJ; that is the product contained 100% FJ—in this case, OJ. This includes 100% FJ made from concentrate and 100% FJ with added nutrients, such as calcium or vitamin D; but does not include juice drinks or other products that contain less than 100% fruit juice. Individual food codes in NHANES 2003-2004 and 2005-2006 were used to determine intake of 100% OJ. Consumers of 100% OJ were defined as those participants consuming any amount of 100% OJ on either day of the 24-hour recalls. To determine nutrient intake, the USDA Food and Nutrient Database for Dietary Studies, versions 2 [38] and 3 [39] were used for NHANES, 2003-2004 and 2005-2006, respectively. Nutrients examined included macronutrients, dietary fiber, and sodium and also those micronutrients likely to be provided by 100% OJ: vitamins A, C, and B6; folate; magnesium; and potassium. Intake from supplements was not considered.

### Food group equivalent intakes and healthy eating index (HEI-2005)

Food group equivalent intakes (formerly called MyPyramid equivalents) were determined using MyPyramid Equivalents Database 2.0; when necessary, intakes for NHANES 2005-2006 were hand matched to similar foods. The HEI-2005 was used to determine diet quality [40]. The SAS code used to calculate HEI-2005 scores was downloaded from the Center for Nutrition Policy and Promotion website [41].

### Physiological measures

Height, weight, and waist circumference (WC) were obtained according to NHANES protocols [42]. Body mass index was calculated as body weight (kilograms) divided by height (meters) squared [43]. For the odds ratio (OR) assessments, overweight/obesity and high waist circumference were determined using the National Heart Lung and Blood Institute (NHLBI) Clinical Guidelines [43]. Systolic (SBP) and diastolic blood pressures (DBP) were determined using the standard NHANES protocol [44] and the mean of all values measured was used. Total cholesterol and high density lipoprotein cholesterol (HDL-C) were determined on non-fasted individuals [45] while low density lipoprotein cholesterol (LDL-C) (46), triacylglycerides [46], blood glucose [47], and insulin [47] were determined on only fasted subjects. Per these protocols, not all individuals may have values for all tests (see tables for sample numbers). Metabolic syndrome was defined using the NHLBI Adult Treatment Panel III criteria [48]; that is having 3 or more of the following risk factors: abdominal obesity, WC >102 cm (males), >88 cm (females); hypertension, SBP ≥130 mmHg or DBP ≥85 mmHg or taking anti-hypertensive medications; HDL-C, <40 mg/dL (males), <50 mg/dL (females); high triacylglycerides, ≥150 mg/dL or taking anti-hyperlipidemic medications; high fasting glucose, ≥110 mg/dL or taking insulin or other hypoglycemic agents.

### Statistical analyses

Sampling weights and the sampling units and strata information, as provided by NHANES, were included in all analyses using SUDAAN v10.0 (Research Triangle Institute; Raleigh, NC). Usual intakes were determined using SAS v 9.2 (SAS Institute, Cary, NC). Usual intake determinations represent long term average daily intakes and are determined by removing excessive intra-person variation in intakes; these are the best estimates to compare to dietary recommendations as suggested intakes are to be met over time, rather than measured on a single day. Usual intake of 100% OJ consumption and selected nutrients was calculated using the National Cancer Institute (NCI) method [49]. For UI of 100% OJ, which is consumed episodically, the two part NCI model (probability and amount) was used; for nutrients which are consumed daily by most people, the one part model was used. The NCI SAS macros (Mixtran v1.1 and Distrib v1.1) were used to generate parameter effects after covariate adjustments and to estimate the distribution of usual intake via Monte Carlo simulation methods, respectively [49]. Covariates in this study were day of the week of the 24-hr recall [coded as weekend (Friday-Sunday) or weekday (Monday-Thursday)] and sequence of dietary recall (first or second). Software provided by NCI was used with the two days of intake using one-day sampling weights to obtain appropriate variance estimates. Balanced repeated replication (BRR) was performed to obtain standard errors (SE) and confidence intervals (CI) for the percentiles; BRR weights were constructed with Fay adjustment factor M = 0.3 (perturbation factor 0.7) and further adjusted to match the initial sample weight totals within specific age/gender/ethnicity groupings for the full dataset. The Dietary Reference Intake (DRI) age groups were used to present UI for each of the nutrients studied.

To assess the extent of inadequate intake of vitamins A and C, folate, and magnesium, the Estimated Average Requirements (EAR) cut-point method proposed by the Institute of Medicine [50] was used. The EAR is the appropriate DRI to use when assessing the adequacy of group intakes [50]. The EAR cut-point method provides an estimate of the proportion of individuals in the group with inadequate intakes by age and gender. For nutrients without an EAR, *i.e.* sodium and potassium, the percent above the Adequate Intake (AI) was determined.

To determine if there were significant differences (p < 0.05) for the percentage of 100% OJ consumers vs non-consumers with intakes less than the EAR or above the AI a Z-statistic for differences in population proportions was used. Linear regression was used to determine differences in 100% OJ consumers and non-consumers for physiological measures. Logistic regression was used to determine if 100% OJ consumers had a lower OR of being overweight or obese or had other health risk factors. Covariates for linear and logistic regression included energy (kcals), age, gender, ethnicity, poverty index ratio, and physical activity for body weight and BMI; for other physiological measures BMI was also added as a covariate. Physical activity was determined from physical activity questionnaires and separated subjects into three categories: sedentary, moderate activity, and vigorous activity [51].

## Results

### Usual intake of orange juice

The sample consisted of adults 19 years of age and older (n = 8,861) of which 2,310 (23.8%) consumed 100% OJ. Per capita consumption was 50.3 ± 1.8 ml/day, whereas among consumers, consumption was 210.0 ± 3.8 ml/day. Per capita consumption and consumption among consumers was higher in males (p < 0.05) than in females. The 75^th^ percentile among consumers was 259.3 ± 6.8 ml/day (Table 1).

**Table 1:** Usual intake of orange juice (ml/d) in the total population and consumers

	**Total population**		**Consumers only**	**Percentiles of intake among consumers**		
	**N = 8,861**		**n = 2,310**			
Gender	Mean ± SE	Pct.	Mean ± SE	25th ± SE	50th ± SE	75th ± SE
Combined	50.3 ± 1.8	23.8	210.0 ± 3.8	109.4 ± 3.0	468.6 ± 4.7	259.3 ± 6.8
Male	59.2 ± 2.7^a^	24.5	235.6 ± 5.3^a^	118.3 ± 4.1	192.2 ± 2.0	286.9 ± 8.3
Female	41.4 ± 2.1^b^	23.1	177.4 ± 5.0^b^	94.6 ± 3.8	147.9 ± 7.4	227.7 ± 7.7

Data source: Adults 19+ years of age participating in NHANES 2003-2006 with consumers defined as orange juice consumption on either of two days of intake assessment.

Means with different letters indicate a significant difference between genders p < 0.05.

1 ml = 0.0338 US fluid oz.

### Usual intake of macronutrients, and selected micronutrients

The UI of carbohydrates, total sugars, and dietary fiber was higher (p < 0.05) in consumers than in non-consumers (Table 2). Table 3 shows that in 100% OJ consumers, the UI of vitamins A, B6, and C; folate; and magnesium was higher (p < 0.05) than non-consumers, and consumers were less likely to be below the EAR than non consumers (p < 0.05) (Table 3). Those consuming 100% OJ had a usual mean intake of vitamin A of 660 ± 15 Retinol Activity Equivalents (RAE) μg/d compared with 580 ± 8 RAE μg/d; approximately 40% of those consuming 100% OJ were below the EAR for vitamin A, compared with 54% of non-consumers (both p < 0.05). Orange juice consumers had a usual mean intake of folate of 606 ± 10 Dietary Folate Equivalents (DFE) μg/d compared with 521 ± 6 DFE μg/d; approximately 6% of 100% OJ consumers were below the EAR, compared with 15% for non-consumers (both p < 0.05). Adults consuming 100% OJ had a mean usual intake of vitamin C of 146 ± 2.4 mg/d compared with approximately 67 ± 1.3 mg/d for non-consumers. On average, 100% OJ consumers were not below the EAR, compared with 59% of non-consumers (p < 0.05). Adults that consumed 100% OJ had higher usual mean intake of 313 ± 4 mg/d magnesium compared with 283 ± 3 mg/d for non-consumers. Approximately 64% of adults that consumed 100% OJ were below the EAR, compared with 52% for non-consumers (both p < 0.05). The usual mean intake of potassium of 100% OJ consumers was 3026 ± 36 mg/d, compared with 2623 ± 22 mg/d for non-consumers; approximately 4% of 100% OJ consumers were above the AI, compared with only 2% of non-consumers (p < 0.05).

**Table 2:** Energy and macronutrient usual intakes among consumers and non-consumers of orange juice

	**Usual intake**	**Percentile**				
**Group**	**Mean ± SE**	**10**	**25**	**50**	**75**	**90**
Energy, Kcal/d						
Consumer	2248 ± 33	1400	1702	2126	2684	3569
Non-Consumer	2185 ± 15	1339	1656	2087	2622	3170
Protein, g/d						
Consumer	84.9 ± 1.3	52.2	62.5	79.8	102.0	125.3
Non-Consumer	83.4 ± 0.7	50.4	62.8	80.0	100.6	121.4
Carbohydrates, g/d						
Consumer	279 ± 4^a^	178	214	265	330	402
Non-Consumer	260 ± 2^b^	155	194	248	314	384
Total sugars, g/d						
Consumer	133 ± 2.3^a^	77.4	96.9	124	160	201
Non-Consumer	119 ± 1.3^b^	57.2	79.0	110	149	192
Dietary fiber, g/d						
Consumer	16.6 ± 0.3^a^	9.6	12.2	15.8	20.1	24.7
Non-Consumer	15.3 ± 0.3^b^	8.6	11.1	14.5	18.5	22.9
Total fat, g/d						
Consumer	83.1 ± 1.4	47.7	60.8	78.7	101.2	124.6
Non-Consumer	83.7 ± 0.7	47.6	61.3	79.9	102.1	125.1
Saturated fatty acids, g/d						
Consumer	27.4 ± 0.6	15.2	19.6	25.8	33.4	41.6
Non-Consumer	27.8 ± 0.3	15.0	19.7	26.3	34.3	42.8

Data source: Adults 19+ years of age participating in NHANES 2003-2006 with consumers defined as orange juice consumption on either of two days of intake assessment.

n: 2,310 OJ consumers and 6,551 non-consumers.

Means with different letters are significantly different, p < 0.05.

**Table 3:** Selected micronutrient usual intakes among consumers and non-consumers of orange juice and comparison to Estimated Average Requirements (EAR) or Adequate Intake (AI)

	**Usual intake**	**Percentile**					**EAR**
**Group**	**Mean ± SE**	**10**	**25**	**50**	**75**	**90**	**% Below ± SE**
Vitamin A, RAE ug/d							
Consumer	660 ± 15^a^	364	471	618	802	1009	39.7 ± 2.5^a^
Non-Consumer	580 ± 8^b^	265	373	529	730	960	54.0 ± 1.2^b^
Vitamin B-6, mg/d							
Consumer	2.1 ± 0.0^a^	1.3	1.6	2.0	2.6	3.2	9.5 ± 1.0^a^
Non-Consumer	1.9 ± 0.0^b^	1.1	1.4	1.8	2.3	2.9	16.7 ± 1.4^b^
Folate, DFE ug/d							
Consumer	606 ± 10^a^	358	451	578	730	890	5.8 ± 0.7^a^
Non-Consumer	521 ± 6^b^	288	371	487	634	796	15.1 ± 0.9^b^
Vitamin C, mg/d							
Consumer	146 ± 2.4^a^	110	125	148	167	178	0.0 ± 0.0^a^
Non-Consumer	66.6 ± 1.3^b^	26.1	38.9	58.5	85.4	117.1	59.0 ± 1.4^b^
Magnesium, mg/d							
Consumer	313 ± 4^a^	193	238	296	372	449	51.6 ± 1.6^a^
Non-Consumer	283 ± 3^b^	170	213	270	339	411	63.7 ± 1.2^b^
	Usual Intake	Percentile					AI
Group	Mean ± SE	10	25	50	75	90	% Above ± SE
Sodium, mg/d							
Consumer	3483 ± 53	2066	2604	3338	4213	5107	98.4 ± 0.3
Non-Consumer	3501 ± 29	2137	2654	3351	4194	5074	98.8 ± 0.2
Potassium, mg/d							
Consumer	3026 ± 36^a^	1978	2396	2939	3564	4195	4.1 ± 0.8^a^
Non-Consumer	2623 ± 22^b^	1610	2009	2532	3140	3756	1.8 ± 0.2^b^

Data source: Adults 19+ years of age participating in NHANES 2003-2006 with consumers defined as orange juice consumption on either of two days of intake assessment.

n: 2,310 OJ consumers and 6,551 non-consumers.

Means with different letters are significantly different, p < 0.05.

### Diet quality and food group equivalents usual intake

Diet quality, as measured by HEI-2005, was significantly higher (p < 0.05) in consumers than in non-consumers (55.0 ± 0.4 vs 49.7 ± 0.3) (Table 4). Total fruit (1.8 ± 0.05 vs 0.7 ± 0.02 cup equivalents/d), fruit from juice (1.1 ± 0.03 vs 0.2 ± 0.01 cup equivalents/d) and whole fruit (0.7 ± 0.03 vs 0.5 ± 0.02 cup equivalents/d) were all higher for consumers as compared to non-consumers. In addition, whole grain consumption was higher (p < 0.05) in consumers (0.8 ± 0.03 ounce equivalents) than in non-consumers (0.6 ± 0.02 ounce equivalents).

**Table 4:** Diet quality, as measured by healthy eating index and select usual intakes of MyPyramid food components among consumers and non-consumers of orange juice

	**Usual intake**	**Percentile**				
**Group**	**Mean ± SE**	**10**	**25**	**50**	**75**	**90**
Healthy Eating Index, score						
Consumer	55.0 ± 0.4^a^	44.5	49.3	54.9	60.6	65.7
Non-Consumer	49.7 ± 0.3^b^	38.4	43.5	49.4	55.6	61.4
Total dairy, cup equivalents						
Consumer	1.6 ± 0.05	0.6	0.9	1.4	2.1	2.9
Non-Consumer	1.5 ± 0.002	0.5	0.8	1.3	2.0	2.8
Total fruit, cup equivalents						
Consumer	1.8 ± 0.05^a^	1.1	1.4	1.8	2.1	2.3
Non-Consumer	0.7 ± 0.02^b^	0.2	0.3	0.6	0.9	1.4
Fruit juice, cup equivalents						
Consumer	1.1 ± 0.03^a^	0.8	0.9	1.1	1.3	1.4
Non-Consumer	0.2 ± 0.01^b^	0.0	0.0	0.1	0.2 0.4	
Whole fruit, cup equivalents						
Consumer	0.7 ± 0.03^a^	0.1	0.3	0.6	1.0	1.4
Non-Consumer	0.5 ± 0.02^b^	0.1	0.2	0.4	0.7	1.1
Total grain, ounce equivalents						
Consumer	7.0 ± 0.1	4.2	5.3	6.7	8.4	10.0
Non-Consumer	6.8 ± 0.1	3.8	4.9	6.4	8.3	10.3
Whole grain, ounce equivalents						
Consumer	0.8 ± 0.03^a^	0.2	0.4	0.7	1.0	1.5
Non-Consumer	0.6 ± 0.02^b^	0.1	0.2	0.5	0.9	1.3
Total vegetables, cup equivalents						
Consumer	1.7 ± 0.03	0.9	1.2	1.6	2.0	2.5
Non-Consumer	1.6 ± 0.03	0.9	1.2	1.5	2.0	2.5

Data source: Adults 19 + years of age participating in NHANES 2003-2006 with consumers defined as orange juice consumption on either of two days of intake assessment.

n: 2,310 OJ consumers and 6,551 non-consumers.

Means with different letters are significantly different, p < 0.05.

### Anthropometric and cardiovascular risk factors

Consumers of 100% OJ had a lower mean BMI than non-consumers (27.6 ± 0.18 vs 28.5 ± 0.11 kg/m^2^; p = 0.0001) (Table 5). Adults that consumed 100% OJ also had lower total cholesterol (197.6 ± 1.2 mg/dL v 200.8 ± 0.75 mg/dL; p = 0.0220) and lower LDL-C (112.5 ± 1.4 mg/dL v 116.7 ± 0.93 mg/dL; p = 0.0110) levels than those that did not consume 100% OJ. Serum vitamin C (1.1 ± 0.01 vs 0.9 ± 0.01 mg/dL; p < 0.0001), red blood cell folate (309.3 ± 3.6 vs 285.3 ± 2 ng/ml RBC; p < 0.0001), and serum folate (14.8 ± 0.24 vs 13.7 ± 0.25 ng/ml; p = 0.0013) were higher in consumers of 100% OJ than in non-consumers (Table 5). There were no differences among consumers and non-consumers in waist circumference, SBP or DBP, C-reactive protein, HDL-cholesterol, triacyglycerides, blood glucose, insulin, or homocysteine levels.

**Table 5:** Physiological measures among consumers and non-consumers of orange juice

	**Consumers**		**Non-consumers**		
**Variable**	**n**	**LSM ± SE**	**n**	**LSM ± SE**	**p-Value**
Body Weight^*^ (kg)	2130	81.3 ± 0.20	6150	81.4 ± 0.12	0.5072
BMI^*^ (kg/m^2^)	2130	27.6 ± 0.18	6150	28.5 ± 0.11	<0.0001
Waist Circumference^*^ (cm)	2069	97.1 ± 0.17	6005	97.3 ± 0.10	0.4381
Systolic Blood Pressure (mmHg)^**^	1801	124.0 ± 0.53	5296	123.8 ± 0.30	0.7604
Diastolic Blood Pressure (mmHg)^**^	1801	70.8 ± 0.42	5296	71.3 ± 0.23	0.2976
Serum Vitamin C (mg/dL)^**^	2022	1.1 ± 0.01	5813	0.91 ± 0.01	<0.0001
C-Reactive Protein^**^ (mg/dL)	2041	0.43 ± 0.03	5874	0.41 ± 0.01	0.5922
Total Cholesterol^**^ (mg/dL)	2034	197.6 ± 1.2	5857	200.8 ± 0.75	0.0220
HDL-Cholesterol^**^ (mg/dL)	2033	53.7 ± 0.45	5857	53.8 ± 0.24	0.8230
Triglycerides^**^ (mg/dL)	954	141.6 ± 3.6	2833	147.4 ± 3.3	0.2246
LDL-Cholesterol^**^ (mg/dL)	939	112.5 ± 1.4	2746	116.7 ± 0.93	0.0110
Plasma Glucose^**^ (mg/dL)	960	103.0 ± 1.4	2856	102.4 ± 0.62	0.6853
Insulin^**^ (uU/mL)	951	11.6 ± 0.38	2823	11.3 ± 0.25	0.4441
RBC Folate^**^ (ng/mL RBC)	2044	309.3 ± 3.6	5872	285.3 ± 2.0	<0.0001
Serum Folate^**^ (ng/mL)	2037	14.8 ± 0.24	5849	13.7 ± 0.25	0.0013
Homocysteine^**^ (umol/L)	1988	8.7 ± 0.08	5729	8.9 ± 0.06	0.0853

^*^Adjusted for energy (kcal), age, gender, ethnicity, poverty income ratio, and physical activity.

^**^Adjusted for energy (kcal), age, gender, ethnicity, poverty income ratio, BMI, and physical activity.

Abbreviations: LSM = least square mean; SE = standard error; BMI = body mass index; HDL-C = high density lipoprotein-cholesterol; LDL-C = low-density lipoprotein-cholesterol; RBC = red blood cell.

### Risk of metabolic syndrome and risk factors for metabolic syndrome

Males that consumed 100% OJ showed a 36% reduced risk [OR: 0.62; 95^th^ CI: 0.45-0.91] of MetS; no differences were observed in females (OR: 1.41 95^th^ CI: 0.96-2.07) (Table 6). Male consumers of 100% OJ also showed a 23% reduced risk (OR: 0.77 95^th^ CI: 0.61-0.99) of low HDL-C levels. Overall there was a 21% reduced risk (OR: 0.79; 95^th^ CI: 0.65-0.95) of obesity in adults that consumed 100% OJ compared with non-consumers.

**Table 6:** Risk of metabolic syndrome, increased risk of individual metabolic syndrome components and other health factors among adult (19+ yrs) consumers and non-consumers of orange juice

**Risk**	**OR ± SE**	**LCL, UCL**	**p-Value**	**OR ± SE**	**LCL, UCL**	**p-Value**	**OR ± SE**	**LCL, UCL**	**p-Value**
	**All**			**Female**			**Male**		
MetS	0.93 **±** 0.13	0.71, 1.22	0.5790	1.41 **±** 0.28	0.96, 2.07	0.0795	0.64 **±** 0.12	0.45,0.91	0.0119
Elevated BP	0.98 ± 0.07	0.85, 1.13	0.7586	0.98 **±** 0.15	0.73, 1.31	0.8948	0.95 **±** 0.12	0.75, 1.22	0.7078
High Glucose	0.96 **±** 0.10	0.78, 1.18	0.6772	1.08 **±** 0.17	0.79, 1.47	0.6180	0.82 **±** 0.11	0.64, 1.06	0.1361
High TG	1.11 **±** 0.13	0.89, 1.39	0.3575	1.41 **±** 0.25	1.00, 1.99	0.0503	0.91 **±** 0.11	0.72, 1.15	0.4474
Elevated WC	0.98 **±** 0.15	0.73, 1.33	0.9067	1.09 **±** 0.24	0.72, 1.66	0.6890	0.83 **±** 0.15	0.59, 1.18	0.3052
Low HDL-C	0.92 **±** 0.08	0.78, 1.09	0.3518	1.08 **±** 0.11	0.89 1.31	0.4271	0.77 **±** 0.10	0.61, 0.99	0.0406
Obese	0.79 **±** 0.08	0.65, 0.95	0.0116	0.76 **±** 0.10	0.59, 0.97	0.0289	0.79 **±** 0.09	0.64, 0.97	0.0276
Overweight	1.13 **±** 0.07	0.99, 1.28	0.0699	1.18 **±** 0.10	0.99, 1.39	0.0581	1.07 **±** 0.09	0.91, 1.26	0.3976
Overweight or Obese	0.89 **±** 0.07	0.76, 1.04	0.1437	0.88 **±** 0.09	0.73, 1.08	0.2216	0.85 **±** 0.09	0.69, 1.06	0.1461
High LDL-C	0.82 **±** 0.10	0.66, 1.03	0.0908	0.76 **±** 0.12	0.57, 1.03	0.0783	0.85 **±** 0.14	0.63, 1.16	0.3163

* Reference group: Non-consumers of orange juice with odds ratio set at 1.0.

All Metabolic Syndrome Components: Elevated Waist Circumference ≥102 cm in men or ≥88 cm in women; Elevated Triglycerides ≥150 mg/dL or taking medication for Elevated Triglycerides (Antihyperlipidemic Agents or Nicotinic Acid Derivatives); Reduced HDL-C <40 mg/dL in men or <50 mg/dL in women or taking medication for Reduced HDL-C (Antihyperlipidemic Agents or Nicotinic Acid Derivatives); Elevated BP ≥130 mmHg Systolic or ≥85 mmHg Diastolic or taking medication for Elevated BP (Antihypertensive Combinations); Elevated Fasting Glucose ≥100 mg/dL or taking medication for Elevated Glucose (Antidiabetic Agents); Metabolic Syndrome (≥3 risk factors above). Other risk factors: Elevated LDL-C ≥100 mg/dL; Overweight BMI ≥25 and <30; Obese BMI ≥30; Overweight or Obese BMI ≥25.

Abbreviations: OR = odds Ratio; LCL = lower confidence level; UCL = upper confidence level; SE = standard error; MetS = metabolic syndrome; BP = blood pressure; TG = triglycerides; WC = waist circumference; HDL-C = high density lipoprotein-cholesterol; LDL-C = low density lipoprotein-cholesterol.

## Discussion

Approximately 24% of the population consumed 100% OJ on either of the days when a 24 hour recall was taken. Males consumed more 100% OJ, both as a percentage of consumers and in amount. The percent of consumers was similar to that of children [52]. *Per capita* UI consumption was 50.3 ml/d; however the UI for consumers was 210.0 ml/d. Unlike children, where there is a specific recommendation for consumption of 100% FJ [53], there is no recommendation for consumption of 100% FJ by adults, other than “the majority of the fruit recommended should come from whole fruits, including fresh, canned, frozen, and dried forms, rather than from juice” [19].

The rationale for limiting 100% FJ intake is that it lacks fiber and can contribute to excess energy consumption when consumed in excess [19]. A modeling study, commissioned by the 2005 Dietary Guidelines Advisory Committee [54] suggested that dietary fiber was lower when whole fruit was removed from the diet, which led to the recommendation that intake of no more than one-third of fruit servings should come from 100% FJ and two-thirds should come from whole fruit. However, this study and others [1-4,52] have shown that either consumers of 100% FJ had higher intakes of dietary fiber than non-consumers or there was no difference in fiber consumption between the groups. Since 100% FJ is low in dietary fiber, it suggests that other higher fiber foods, including whole fruit, are consumed by consumers of 100% FJ; this was shown not only in this study of 100% OJ consumers, but has been shown in other studies as well [1,2,52].

As expected, 100% OJ consumers had increased intake of nutrients typically found in 100% OJ (i.e*.* vitamin C, folate, and potassium). Consumers were also less likely to have intakes below the EAR for vitamins A, B-6, and C; folate; and magnesium than non-consumers. The reduction in the percentage of the population with inadequate intakes of these nutrients associated with 100% OJ consumption indicates the value of consuming a nutrient dense beverage [17]. Mean potassium UI was also higher in consumers than non-consumers and the percentage of the population above the AI was higher. This is an important finding since potassium was identified as a nutrient of public health concern [19]. To our knowledge this is the first report studying the association between the consumption of 100% OJ and nutrient adequacy in adults using the recommended UI procedures.

Diet quality, as measured by HEI-2005, was approximately 10% higher in 100% OJ consumers. While the increase was due in part to the increase in whole fruit and FJ consumption, consumers also had a higher UI of whole grains. Although intake of total fruit, whole fruit, and FJ was higher in 100% OJ consumers, overall intake from the fruit food groups was low. Despite extensive, coordinated public health campaigns by government, industry, and others [55], fruit consumption in adults remains low [56]. Since a 236.6 ml serving of 100% OJ counts as part of the recommendation for the fruit group, moderate consumption of 100% OJ can help individuals meet fruit intake recommendations.

The potential association of consumption of 100% FJ and weight in children has been debated in the literature for more than a decade [1,2,5-8,57-62]; however, less is known about this relationship in adults. Participants in the Nurses’ Health Study II with a higher consumption of 100% FJ had a larger weight gain than those with lower fruit 100% FJ consumption, although the amounts and types of 100% FJ consumed, and specific covariates used in the analyses, were not clear [11]. Another study [9] showed that self reported BMI was lower in consumers of 100% FJ. Ours was the first study that used a nationally representative adult population that showed consumers of 100% OJ had a lower BMI than non-consumers. These findings are important since 100% OJ has the highest *per capita* consumption [16] among the juices and therefore has the potential to be an important component of the diet. Clinical studies that incorporated high levels of 100% OJ (750 ml [24] or 500 ml [30]) as an intervention have reported no increases in weight or other anthropometric measures over the course of the study.

Total cholesterol levels and LDL-C levels were both significantly lower in consumers of 100% OJ than non-consumers. Compounds found in 100% OJ, including hesperidin, naringin, or limonoids or their circulating aglycone forms, have been shown to lower total or LDL-C in animal models [63,64]. It was hypothesized that these compounds may have inhibited 3-Hydroxy-3-methyl-glutaryl coenzyme A reductase and increased the expression of LDL-C receptors in the liver, a mechanism similar to statins. These compounds have also been shown to reduce the net secretion of apolipoprotein B, which in turn may help inhibit cholesterol ester synthesis [20,65].

Orange juice, at higher intake amounts (750 ml) has also been shown to lower LDL-C and raise HDL-C in a randomized clinical trial of hypercholesterolemia individuals [24]. Although the present study did not look separately at individuals with hypercholesterolemia, it did show that a more realistic consumption of 100% OJ was associated with reduced total cholesterol and LDL-C levels. It is not clear why there was no difference shown between HDL-C levels between 100% OJ consumers and non-consumers, as may have been suggested by clinical trials; the response may be dose-dependent or dependent on continual consumption. There was a 23% lower risk of low HDL-C levels in males only.

Consumption of 100% OJ was associated with a 21% lower risk of obesity in men and women. This was similar to the findings of Pereira and Fulgoni [10] that looked at the risk of obesity and consumption of 100% FJ in participants of NHANES 1999-2004. They also showed a significantly lower risk of metabolic syndrome, whereas this study showed a lower risk in males only. That study showed a much higher intake of 100% FJ, compared with the intake of 100% OJ only; but there were also other differences in the population, since they showed, for example that consumers were more likely to be female. Our study showed that 100% OJ consumers were more likely to be males. Consumption differences of 100% FJ in adults need to be studied further.

Strengths of this study include that it encompassed a large nationally representative sample achieved through combining several sets of NHANES data releases. The study also uses the NCI method to assess UI and the percentage of the population below recommended levels in 100% OJ consumers and non-consumers, as well as adjustment for numerous covariates including physical activity.

Twenty-four hour dietary recalls have several inherent limitations. Participants relied on memory to self-report dietary intakes; therefore, data were subject to non-sampling errors, including underreporting of energy and examiner effects. Respondents may not have differentiated between 100% OJ or a fruit drink/ade. Confusion over these beverages has been reflected in several studies that assessed a combined 100% FJ and juice drink or sweetened FJ category [66-69]. The use of AI cannot be used to determine the prevalence of inadequate intake in a group. Rather, if the mean intake of a group is at or above the AI, and the variance of intake in the group of interest is similar to the variance of intake used in the population originally used to set the AI, the prevalence of inadequate nutrient intakes is likely to be low [50]. Finally, since causal inferences cannot be drawn from NHANES analyses, and due to multi-collinearity of diet, foods other than 100% OJ may have contributed to differences in nutrient intake of the participants.

## Conclusions

Consumption of 100% OJ was associated with better diet quality and an increased prevalence of meeting the EAR for key nutrients and other biomarkers of positive health outcomes, including lower total cholesterol and LDL levels. Consumers of 100% OJ had lower mean BMI and a decreased risk of obesity. In addition, males had a decreased risk of metabolic syndrome. These results suggested that 100% OJ consumption should be encouraged as a component of a healthy diet to help individuals meet nutrient and fruit intake recommendations.

## Abbreviations

AI: Adequate intake; BMI: Body mass index; BRR: Balanced repeated replication; CI: Confidence interval; DBP: Diastolic blood pressure; DFE: Dietary folate equivalents; DRI: Dietary reference intake; EAR: Estimated average requirements; FJ: 100% Fruit juice; HDL-C: High density lipoprotein-cholesterol; HEI-2005: Healthy eating index-2005; LDL-C: Low density lipoprotein-cholesterol; MetS: Metabolic syndrome; NHANES: National health and Nutrition examination survey; NHLBI: National heart, lung, and blood institute; OJ: 100% Orange juice; OR: Odds ratio; RAE: Retinol activity equivalents; SBP: Systolic blood pressure; SFA: Saturated fatty acids; UI: Usual intake; WC: Waist circumference.

## Competing interests

Gail Rampersaud’s position at the University of Florida is co-funded by the Florida Department of Citrus. None of the other authors declare a competing interest.

## Authors’ contributions

All authors contributed equally to this work. All authors read and approved the final manuscript.
